# Study of the efficacy and safety of various pharmacotherapy regimens for atypical depression in the framework of bipolar affective disorder, recurrent depressive disorder, psychogenic depression

**DOI:** 10.1192/j.eurpsy.2022.1448

**Published:** 2022-09-01

**Authors:** M. Verbitskaya, N. Tyuvina, Y. Tyulpin

**Affiliations:** Sechenov First Moscow State Medical University of the Ministry of Health of Russia (Sechenov University), Department Of Psychiatry And Narcology, Clinic Of Psychiatry And Narcology Named After S.s. Korsakov, Moscow, Russian Federation

**Keywords:** recurrent depressive disorder, psychogenic depression, Bipolar Affective Disorder, atypical depression

## Abstract

**Introduction:**

To improve the effectiveness of treatment for atypical depression, it is necessary to revise the accumulated experience, taking into account new knowledge and drugs.

**Objectives:**

Comparative study of the efficacy and safety of therapy for atypical depression (AtD) in the structure of bipolar affective disorder (BAD), recurrent depressive disorder (RDR) and psychogenic depression (PD).

**Methods:**

Clinical and clinical follow-up methods examined 77 patients with AtD, of which 35 - with bipolar disorder, 18 - with RDR and 24 - with PD. Patients in all three groups received monotherapy with an antidepressant or a mood stabilizer, or a combination of antidepressant and antipsychotic, antidepressant and mood stabilizer, mood stabilizer and antipsychotic, as well as a combination of antidepressant, antipsychotic and mood stabilizer.

**Results:**

Agomelatine was the most frequently used (27.3%) and effective in reducing MADRS in all groups both in monotherapy and in combination with other drugs. Also in the PD group, escitalopram and vortioxetine were highly effective. Of the antipsychotics, when combined with antidepressants, sulpiride was found to be the most effective. When comparing the tolerance of antidepressants in all groups showed the best results (by the CGI scale), agomelatine and venlafaxine, in the BAR group is also vortioxetine.

**Conclusions:**

The best strategy for effective and safe treatment of atypical depression is the use of modern antidepressant, which does not increase the symptoms of the atypical spectrum and, if necessary, can be supplemented with some antipsychotics.

**Disclosure:**

No significant relationships.

